# Pre-Replicative Repair of Oxidized Bases Maintains Fidelity in Mammalian Genomes: The Cowcatcher Role of NEIL1 DNA Glycosylase

**DOI:** 10.3390/genes8070175

**Published:** 2017-06-30

**Authors:** Suganya Rangaswamy, Arvind Pandey, Sankar Mitra, Muralidhar L. Hegde

**Affiliations:** 1 Department of Radiation Oncology, Houston Methodist Research Institute, Houston, TX 77030, USA; srangaswamy@houstonmethodist.org (S.R.); apandey@houstonmethodist.org (A.P.); 2 Weill Cornell Medical College, Cornell University, New York, NY 10065, USA; 3 Houston Methodist Neurological Institute, Houston, TX 77030, USA

**Keywords:** cowcatcher model of pre-replicative repair, post-replicative repair, base excision repair, NEIL1 DNA glycosylase

## Abstract

Genomic fidelity in the humans is continuously challenged by genotoxic reactive oxygen species (ROS) generated both endogenously during metabolic processes, and by exogenous agents. Mispairing of most ROS-induced oxidized base lesions during DNA replication induces mutations. Although bulky base adducts induced by ultraviolet light and other environmental mutagens block replicative DNA polymerases, most oxidized base lesions do not block DNA synthesis. In 8-oxo-G:A mispairs generated by the incorporation of A opposite unrepaired 8-oxo-G, A is removed by MutYH (MYH) for post-replicative repair, and other oxidized base lesions must be repaired prior to replication in order to prevent mutation fixation. Our earlier studies documented S phase-specific overexpression of endonuclease VIII-like 1 (NEIL1) DNA glycosylase (DG), one of five oxidized base excision repair (BER)-initiating enzymes in mammalian cells, and its high affinity for replication fork-mimicking single-stranded (ss)DNA substrates. We recently provided experimental evidence for the role of NEIL1 in replicating-strand repair, and proposed the “cowcatcher” model of pre-replicative BER, where NEIL1’s nonproductive binding to the lesion base in ssDNA template blocks DNA chain elongation, causing fork regression. Repair of the lesion in the then re-annealed duplex is carried out by NEIL1 in association with the DNA replication proteins. In this commentary, we highlight the critical role of pre-replicative BER in preventing mutagenesis, and discuss the distinction between pre-replicative vs. post-replicative BER.

## 1. Introduction

Reactive oxygen species (ROS) generated as by-products of cellular respiration in aerobic organisms and by exogenous genotoxic agents are a major threat to the genome, which is vulnerable to oxidative damage. Transiently formed single-stranded (ss)DNA serving as a template during DNA replication after unwinding of the duplex genome is particularly vulnerable to oxidation by ROS, which induces a plethora of oxidized DNA base lesions and oxidized sugar fragments, as well as DNA strand breaks. The genomic integrity is particularly vulnerable during replication. Most oxidized base lesions will mispair during replication and would not stall a replicative DNA polymerase, causing mutations. In contrast, bulky lesions, which stall replicative polymerases, block replisomes so as to allow repair. However, blocked replication may also lead to fork collapse, causing significant alteration in genomic stability. Furthermore, oxidized deoxynucleotides may be incorporated into the progeny strand during DNA replication. If left unrepaired, these mutations could accumulate in progeny cells—a recipe for pathologies linked to genomic instability, including cancer [1], accelerated aging, and degenerative brain diseases [2]. 

Replication-associated DNA damage thus represents a major challenge to genomic integrity. Mammalian cells have evolved ways to faithfully repair such damage, both in the template strand and post-replicative progeny strand. This involves an intricate collaboration of specific repair machinery with the replication proteins. Repair of most mutagenic base lesions except 8-oxoG (e.g., 5-hydroxyuracil (5-OHU), thymine glycol (TG), hydroxycytosine (5-OHC), formamidopyrimidines (FapyA), 7,8-dihydro-8-oxoadenine (8-oxo-A), uracil glycol) must be carried out prior to replication in order to prevent mutation fixation. How such lesions, that do not block replicative polymerase δ (Polδ), are flagged for pre-replicative repair without causing double-strand breaks (DSBs) was unclear. Our recent study showed that endonuclease VIII-like 1 (NEIL1) DNA glycosylase (DG), a unique base-excision repair (BER)-initiating enzyme in mammalian cells binds to lesion sites in ssDNA substrates in vitro to facilitate fork regression and pre-replicative repair of the damaged base in the re-annealed duplex DNA [3]. In this commentary, we discuss the “cowcatcher” model of pre-replicative DNA repair of oxidized bases that is critical to maintenance of genome fidelity. 

## 2. Key Features of Oxidative Damage Repair in the Mammalian Genome

### 2.1. A Plethora of Oxidized Bases and Their Repair: A Constant Challenge for the Mammalian 

All cellular reactions involving molecular oxygen, in particular mitochondrial respiration, generate O_2_^−^ radicals, H_2_O_2_, and other ROS such as singlet oxygen, OH^−^ radical, and peroxyl, alkoxyl, peroxynitrite, etc. These ROS react with the genome to induce various lesions, including oxidized bases, deoxyribose, abasic apurinic and apyrimidinic (AP) sites, and single-strand breaks (SSBs) with unligatable termini. Most oxidized bases e.g., 5-OHC, 5-OHU, TG, 5-formylU, uracil glycol etc., are mutagenic [4,5], with altered base-pairing properties. The mutagenic potential of oxidized bases widely varies for various lesions, although no definitive data are available about comparative mutagenicity of various base lesions. Their misreplication would cause point mutations whose occurrence in oncogenes and tumor suppressor genes could lead to oncogenic transformation. Significantly higher levels of point mutations observed in many cancer cell genomes underscore the key role of BER in preventing malignancy [6,7]. Oxidatively damaged bases are primarily repaired via the BER pathway, which includes multiple sub-pathways. The complexity of BER arises from diverse factors, including lesion occurrence in replicating vs. nonreplicating DNA, in transcribing vs. non-transcribing DNA, and in the parental vs. nascent DNA strand, together with involvement of several non-canonical proteins and interaction of BER machinery with other repair pathways. 

Several dozen distinct oxidized-base lesions have been identified in the mammalian genome [8]. The most common oxidative guanine lesion 8-oxo-G is generated in vivo at ~2000 lesions per human cell per day [9,10,11,12]. By one estimate, more than 10^5^ base lesions could be induced by endogenous ROS in an aerobic cell genome every day [13]. This enormous damage load may still be an underestimate due to continuous repair, making it difficult to precisely assess the damage in real time. 

### 2.2. Minimal BER Reaction

The minimal essential components of BER include repair-initiating DGs, end-processing enzymes, AP endonuclease (APE1), polynucleotide kinase phosphatase (PNKP), tyrosyl-DNA phosphodiesterase 1 (TDP1) or Aprataxin (APTX), DNA polymerase β (Polβ), nick-sealing DNA ligases (Ligs) 3 or 1, and the scaffold protein X-ray cross-complementing protein (XRCC1). Six oxidized DNA–base-specific DGs identified so far in mammals have been classified based on substrate specificity and their structural motifs. The endonuclease III (Nth) family includes 8-oxoguanine DG (OGG1) and endonuclease III-like 1 (NTHL1). The endonuclease VIII (Nei) family includes endonuclease VIII-like 1–3 (NEIL1, NEIL2, and NEIL3). The sixth member, MutYH (MYH), is an *Escherichia coli* MutY homolog that removes a normal base A opposite 8-oxo-G [14,15]. Another DG, thymine DNA glycosylase (TDG) is important for the removal of T (opposite G) formed by deamination of 5 methylcytosine [16]. Unlike MYH and TDG, the NEILs, OGG1, and NTHL1 have broad substrate specificity and are capable of excising about a half dozen lesions [17,18]. For example, as presented in Table 1, NEIL1 has a preference for ring-opened purines (FapyG, FapyA), TG, 5-OHC, 5-OHU, 5-formylU, and dihydrouracil (DHU). NEIL1 has also been shown to excise G-derived lesion 8-oxo-G in vitro [19] and hydantoin lesions, i.e., guanidinohydantoin (Gh) and spiroiminodihydantoin (Sp), particularly from ssDNA substrates [19,20,21]. While NEIL1 has weak activity for 8-oxoG in duplex DNA substrates, in comparison to OGG1, it is the only DNA glycosylase that can excise 8-oxodG in ss DNA substrates like replication-fork mimicking primer template or bubble substrates, in vitro. However, the in vivo significance of these in vitro observations needs to be established. The AP lyase activity in the Nth family DGs carries out β-elimination to generate 3′-phosphor-α,β-unsaturated aldehyde (3′-PUA) [22] and the NEIL1/2 DGs belonging to the Nei family catalyze βδ-elimination to generate 3′-P terminus after excision of the damaged base [23]. These distinct 3′ blocked termini are then processed by APE1 and PNKP, respectively, to form DNA–polymerase-compatible 3′-OH ends [24]. The 5′-deoxyribosephosphate (5′-dRP) generated by APE1 cleavage of the AP site is removed by intrinsic 5′-dRP lyase activity of DNA Polβ before incorporation of the missing base in the single-nucleotide (SN-) BER reaction, also known as short patch (SP-) BER. Finally, DNA Lig 3, which usually forms a complex with XRCC1, seals the nick in SN-BER. If this 5′-dRP moiety is further oxidized to become resistant to Polβ activity, flap endonuclease 1 (FEN-1) displaces about 2–8 nucleotides containing 5′-dRP, and the gap is filled by proliferating cell nuclear antigen (PCNA)-stimulated Polβ or Polδ/ε, depending on the cell-cycle status, in a long patch (LP-) BER. Other components of LP-BER are replication factor C (RF-C) and Lig 1 [25,26]. 

## 3. Complex Regulation of Mammalian Base Excision Repair 

### 3.1. Preformed BERosome Complexes Regulated by Multiple Posttranslational Modifications 

**Figure 1:**
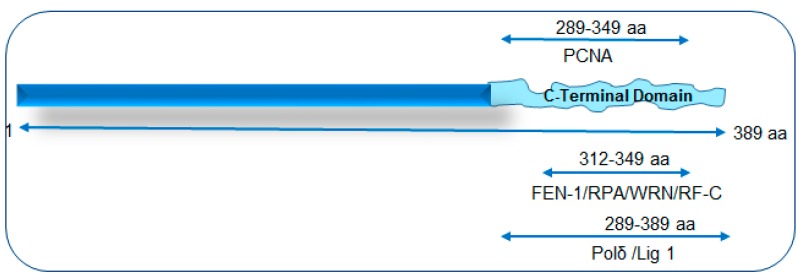
A schematic of NEIL1’s interaction mapping with long patch-base excision repair (LP-BER)/replication proteins to its disordered C-terminal domain. PCNA: proliferating cell nuclear antigen; FEN-1: flap endonuclease 1; RPA: replication protein A; WRN: Werner helicase; RF-C: replication factor C; Polδ: polymerase delta; Lig 1: ligase 1.

Many BER proteins have been reported to undergo post translational modification (PTM), including acetylation, methylation, phosphorylation, SUMOylation and ubiquitination, which provide multidimensional regulation of BER [27]. Most DGs and APE1 have N- or C-terminal disordered, non-conserved regions spanning about 50–150 amino acids (aas). These extensions are absent in their bacterial orthologs and thus appear to have been acquired during evolution, presumably to cope with the challenges of complex repair regulation in mammals (reviewed in Hegde et al. [15]). Furthermore, most PTMs occur on residues within the disordered segments that impact both protein-protein and protein-DNA interactions [28]. OGG1 is phosphorylated to increase its catalytic activity, and is acetylated in response to oxidative stress. Acetylation of NEIL1 is required for chromatin association and for formation of a stable BER complex [29]. In contrast, acetylation of NEIL2 inhibits its activity [30]. Phosphorylation of NEIL11 does not alter the enzyme’s activity in vitro, but is surmised to mediate its interaction with other proteins [31]. The C-terminal region of human NEIL1, spanning about 100 residues, is critical for stabilizing NEIL1’s interaction with replication and other BER proteins (Figure 1). This segment of NEIL1 is also important for efficient repair of the replicating genome [3]). The human NTHL1 truncation mutant lacking the disordered segment of N-terminal 98 aa residues was shown to possess four to five-fold higher catalytic activity [32]. The mammalian uracil DG UNG encodes two splice variants, UNG1 and UNG2, generated by alternative transcription starts, which specifically localize to the mitochondria and nucleus, respectively [33]. Acetylated APE1 has higher AP-site cleavage activity [34], whereas its ubiquitination is required for stability and protein turnover [7,27]. 

### 3.2. DNA Glycosilases May Direct Base Excision Repair Sub-Pathway Choice via Specific Interaction with Downstream Repair Proteins

**Figure 2:**
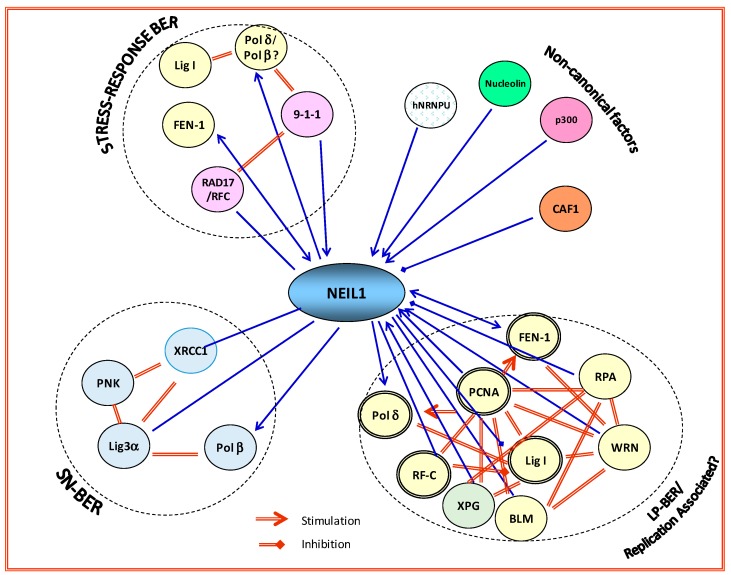
NEIL1 acts as a hub protein in binding to distal partners in BER. NEIL1 forms distinct complexes with single-nucleotide (SN-)BER, long patch (LP-)BER (replication-associated), and other non-canonical proteins, many of which activate its BER activity. RPA inhibits NEIL1 activity in single-stranded (ss)DNA substrates. Binding of nucleolin and p300 are from unpublished data. Polβ: polymerase beta; hNRNP-U: heterogeneous nuclear ribonucleoprotein U; 9-1-1: Rad9–Rad1–Hus1; CAF1: chromatin assembly factor-1; XRCC1: X-ray cross-complementing protein; PNK: polynucleotide kinase; Lig3α: ligase 3 alpha; Polβ: polymerase beta; XPG: Xeroderma pigmentosum Complementation group G; BLM: Bloom syndrome protein.

The protein interaction network, i.e., the interactome, is a common feature of many signaling pathways in mammalian cells. These networks typically involve unstructured, flexible domains in one or more partner proteins that provide a platform for assembling a large dynamic complex. Size fractionation of DNase/RNase-treated HeLa or HEK-293 cell nuclear extracts at physiological ionic strength, showed that a significant fraction of NEIL1, OGG1, and NEIL2 are present in megadalton complexes [3]. Consistently, immunoprecipitates (IPs) of NEIL1 and NEIL2 from human cells contain SN-BER proteins (Polβ, Lig 3α and XRCC1), with which both the NEILs interact in a pairwise fashion, in the absence of DNA [35]. We have also shown NEIL1’s interaction with LP-BER–specific DNA replication proteins, including PCNA, FEN-1, RF-C), Polδ, and DNA Lig 1 [36,37,38,39]. Similarly, APE1 IPs contain Polβ, Lig 3, XRCC1, poly (ADP-ribose) polymerase (PARP1), and TDP1 [40]. More importantly, these IPs of early repair proteins are proficient in complete repair activity in vitro [3,35,41,42]. This suggests that NEIL1 (and possibly other DGs) exist in preformed complexes in vivo, contrary to the prevailing notion of sequential recruitment of BER proteins to the lesion site, followed by hand-off of the intermediate product to the next enzyme in the pathway [43]. In addition, the NEIL1 complex also contains chromatin assembly factor 1 subunit A (CHAF1A), which transiently dissociates after oxidative stress [44]. Based on these findings, it is reasonable to hypothesize that NEIL1 and other DGs may act as hub proteins in regulating formation of the dynamic BER interactome (BERosome), particularly for endogenous oxidized base repair, which enhances repair efficiency. Figure 2 shows NEIL1 interaction with other BER proteins to form distinct repair complexes. However, the formation of large preformed complex(es) raises the issue of stoichiometry and steric interference. Detailed mapping of interaction interfaces among these proteins is vital for unraveling the organization of these complexes. We propose that interaction of DGs with distal repair proteins (such as DNA ligases) is involved in sub-pathway selection. The mammalian BER proteins are thus organized in temporally controlled complexes, presumably for optimum efficiency of damaged-base recognition and repair. Further characterization of such BERosomes under various cellular conditions and cross-talk with other repair/signaling machinery is necessary for gaining insight into the sub-pathway choice. It is important to note that such protein complexes are not unique to BER, and similar situations appear to exist for other DNA excision repair pathways [45,46,47]. 

### 3.3. Individual Dispensability and Overlapping Substrate Specificity of DNA Glycosylases 

The mouse mutants individually lacking OGG1, NTHL1, NEIL1, NEIL2, or MYH [48,49,50,51] or cells derived from these mutants are viable and do not develop a strong phenotype, such as accelerated aging or enhanced incidence of spontaneous cancer. However, deficiency of proteins in later steps of BER including APE1, XRCC1, and Polβ, causes embryonic lethality in mutant mice [52,53]. Genetic variations including single nucleotide polymorphisms (SNPs) in NEIL1 and 2 have been reported to affect their functions [54,55], likely leading to carcinogenesis [56,57,58,59] and other disorders [60,61], as summarized in Table 2. Deficiency in other repair pathways, e.g., nucleotide excision repair (NER) or mismatch repair (MMR), also enhances cancer susceptibility significantly [8,62]. Due to continuous generation of mutagenic and potentially carcinogenic base lesions, whose repair is essential [63], the lack of a strong phenotype in a DG-deficient mouse was unexpected, but led to the assumption that the DGs back up each other for repair initiation. This was supported by the observation of strong increases in cancer susceptibility after combined deficiency of two DGs. For example, mice lacking both OGG1 and MYH or NEIL1 and NTHL1 show strong cancer susceptibility [64,65]. The redundancy among DGs is consistent with their broad and overlapping substrate range. The five DGs account for nearly 30 different oxidized base lesions in the mammalian genome. For example, in addition to its primary substrate, 8-oxo-G, OGG1 also excises ring-opened purine FapyG. The NEILs, initially shown to excise oxidized pyrimidines such as 5-OHU, 5-OHC, and TG, were later shown to excise 8-oxo-G and hydantoins [66]. NTHL1 efficiently repairs oxidized pyrimidines 5-OHU, 5-OHC, 5-formylC, etc. Damage recognition and lesion excision by a DG involves extra-helical flipping of the base into its catalytic pocket and, thus, a DG’s specificity depends on the lesion fit with the binding pocket [67,68]. However, it is likely that plasticity of DGs’ catalytic pockets allows induced fit of diverse substrates. Furthermore, the low turnover of most DGs is consistent with their broad substrate specificity, presumably a price paid for their promiscuity. The DGs’ substrate affinity is also influenced by the location of the damage in transcriptionally active or inactive sequences [69]. DGs and their preferred substrates are listed in Table 1. 

## 4. Replicating and Transcribing DNA Employ Distinct Base Excision Repair Sub-Pathways

The unfolded chromatin at the replication fork or in the transcription bubble is more prone to oxidative damage than condensed chromatin [72,73]. Unlike bulky adducts, which block replication or transcription to activate NER, most oxidized base lesions do not inhibit DNA or RNA synthesis (reviewed in [74]). Replication of unrepaired base lesions is invariably mutagenic, and their transcription could produce mutant proteins, which could be inactive and/or toxic. Thus, there is an urgency to recognize and repair these lesions prior to all DNA transactions. Recent studies by ourselves and others have suggested that there are distinct BER sub-pathways for repairing transcribing vs. inactive genomes, as well as quiescent vs. replicating genomes [38,75,76,77]. Among the DGs, the NEILs are active on ssDNA, including bubble, and fork-structured DNA substrates that mimic transcription and DNA replication intermediates, respectively [78]. They also have preference for telomeric and promoter *G*-quadruplex *DNA* [79,80], whereas OGG1 and NTHL1, are active only on duplex DNA. However, only NEIL1 is induced during the S phase. NEIL1’s functional interaction with DNA replication proteins is consistent with its role in replication associated (RA)-BER [36,37,81] (Table 3). Hazra and colleagues in collaboration with us characterized the involvement of NEIL2 in transcription associated (TC)-BER where NEIL2 functionally interacts with RNA polymerase II [76]. We provided multiple lines of evidence of support for NEIL1’s role in RA-BER, which are discussed below in detail.

### 4.1. Replication-Associated Base Excision Repair Is Critical for Preventing Mutations in Cycling Cells 

DNA replication in the mammalian genome is initiated at multiple replication origins and proceeds bidirectionally [84,85,86]. The binding of initiator proteins to recognition sequences activates DNA unwinding by helicases as part of the pre-replicative complex [87,88] and promotes the assembly of the multi-enzyme replication complex. Activation of the pre-replicative complex is regulated by cyclin-dependent kinases and other signaling proteins to promote the loading of DNA polymerases and the activation of minichromosome maintenance (MCM) protein helicase [85,89,90]. It is important to note that many proteins from the replication complex have dual roles in repair and DNA replication [85]. Sequence fidelity of both strands of DNA is essential for maintaining genomic integrity in replicating cells, unlike the situation in nonreplicating, terminally differentiated cells, such as neurons, where only the transcribing strand of functional genes is critical [91]. 

### 4.2. Pre-Replicative vs. Post-Replicative Base Excision Repair

The post-replicative repair of an 8-oxo-G:A mispair generated by the incorporation of A opposite 8-oxo-G in the template strand or a U:A pair generated due to incorporation of U opposite A during replication, were previously described [92,93]. Such progeny–strand-specific post-replicative repair has been shown to be tightly coupled to the replication machinery, and it does utilize the proteins involved in replication to repair the lesion. Both UNG2 and MYH associate with PCNA at the replication foci [94]. Thus, MYH and UNG2 interact with the replication machinery, analogous to the classical MMR pathway, targeting the nascent DNA for post-replicative repair [95]. Such coordination between DNA replication and repair ensures that the DNA replication-associated proteins are co-opted during S phase to carry out repair synthesis following excision of an oxidized base [36,77]. Recently it was shown that MYH, UNG2, MPG, NTHL1, NEIL1, 2 and 3 recognize a broad spectrum of oxidized DNA base lesions on nascent DNA for RA-BER [96]. 

However, as mentioned before, unlike U that is misincorporated into the progeny strand, U generated in the template strand due to deamination of C must be repaired prior to replication to prevent fixation of the mutation. Furthermore, removal of A in the template opposite 8-oxo-G in the nascent strand by MYH would be mutagenic, and we previously suggested a distinct alternative repair process involving excision of 8-oxo-G from the nascent strand [97]. Similarly, most other oxidative lesions, including 5-OHU, TG, and 5-OHC, which are major substrates of NEILs, must be repaired pre-replication in the template strand to maintain genomic integrity. 

## 5. Cowcatcher Model of Pre-Replicative Repair: Molecular Insights into Template Strand Repair at the Replication Fork

**Figure 3:**
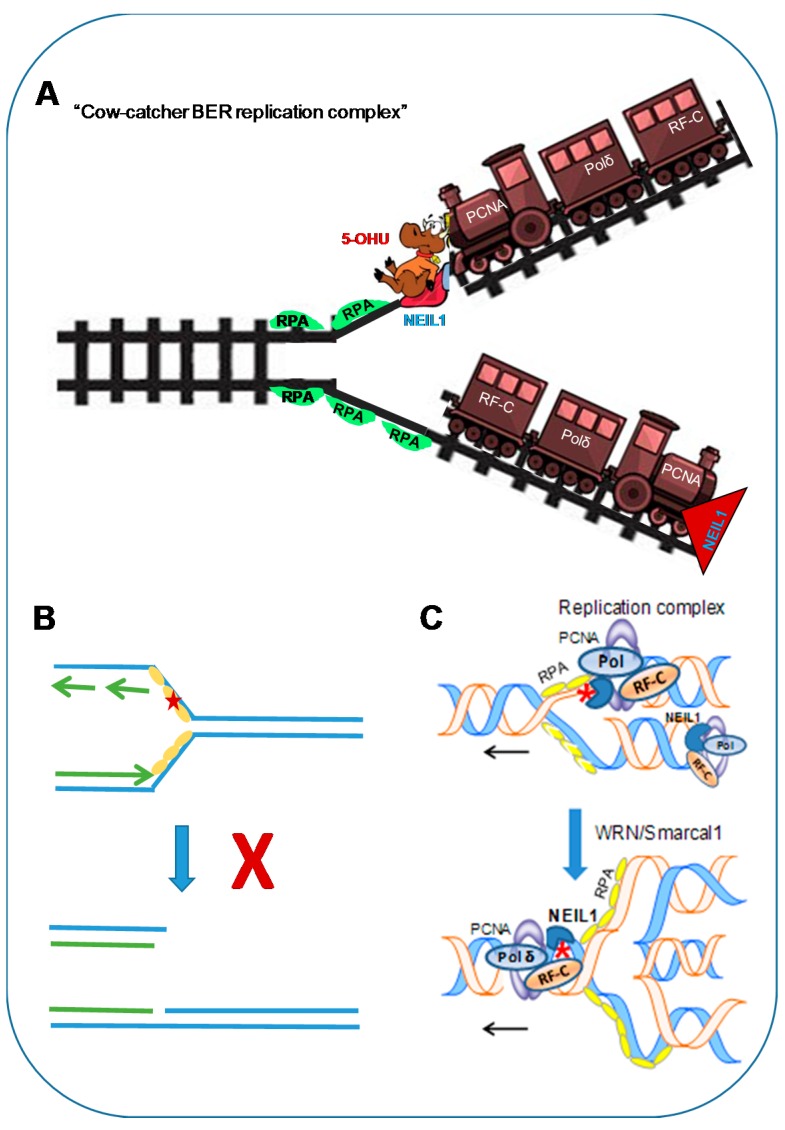
(**A**) ‘Cowcatcher’ model of pre-replicative repair. NEIL1 as part of the replication machinery surveils base lesions in template DNA. (**B**) Its binding to lesion site in RPA-bound ssDNA without removing the lesion is a critical step which would prevent double-stranded break (DSB) formation. (**C**) The resulting stalling and regression of the fork brings the lesion in the re-annealed duplex for faithful repair. 5-OHU: 5-hydroxyuracil; Smarcal1: SWI/SNF related, matrix-associated, actin-dependent, regulator of chromatin, subfamily A-like 1.

We recently provided direct evidence for NEIL1’s ability to repair oxidized bases in the template strand of the replication fork via a complex mechanism that we named the ‘cowcatcher’ model in a simplistic analogy to this exquisitely regulated process [38] that compares it to the structure on the front of an early steam locomotive that served to clear animals or debris from the track ahead of the train (Figure 3). If we visualize the unwinding of DNA at the replication fork analogous to the opening of a zipper, the exposed ssDNA is more susceptible to ROS attack than the duplex DNA. The S–phase-specific induction of NEIL1, together with its stable physical and functional association with several proteins in the DNA replication complex [41,82], suggests that NEIL1 is part of the replication complex, and acts as a cowcatcher at the leading edge of the advancing replication complex carrying out surveillance of oxidized bases in the replicating template. The key features of this model are the ability of NEIL1 to recognize base lesions in ssDNA templates and its nonproductive binding to lesions in ssDNA, which would prevent DSB formation, but signal the stalling of the replication fork, followed by repair in the re-annealed duplex. 

### 5.1. Damage Recognition in single-stranded DNA Template Strand to Stall Replication 

As NEIL1 encounters the base damage in the template strand, it binds to the lesion to flag it, and replication cannot continue. However, binding of the ssDNA template by mammalian ssDNA-binding protein replication protein A (RPA) at the replication fork prevents NEIL1-mediated lesion excision via direct interaction [81]. This is a critical step in pre-replicative BER because if NEIL1 excises oxidized bases and cleaves the template strand in replicating DNA, a one-ended DSB will be generated. Furthermore, we observed that NEIL1 inhibits primer elongation of the RPA-coated template by Polδ in vitro reaction, thereby stalling the replication complex at the lesion site. In addition to lesions like 5-OHU or 5-OHC, which absolutely requires NEIL1’s binding to stall replication, bulkier lesions such as spiro- and imino-hydantoins or TG were shown to block replication. However, as these are NEIL1 substrates, NEIL1 binding might ensure that misincorporation does not occur prior to their repair. The role of NEIL1 in repairing these replication-blocking lesions was indicated by the inhibition of DNA chain growth in oxidatively stressed NEIL1-deficient cells [38].

### 5.2. Pre-Replicative Repair in the Re-Annealed Duplex in the Regressed Fork Structure

Stalling of replication is expected to cause regression of the replication fork, causing fork collapse into a chicken-foot-like structure. This would involve re-annealing of the unwound region spanning the lesion by a helicase such as SMARCAL1 or Werner helicase (WRN) [38] to bring the lesion back in to the duplex state. NEIL1 would then repair the lesion by co-opting proteins of the replication machinery, including PCNA, RF-C, Polδ, FEN-1, and Lig 1. Consistently, NEIL1 is activated by PCNA, RF-C, and FEN-1 [3]. NEIL1 also functionally associates with WRN [98], presumably to coordinate fork regression or resolution (Table 3). Our unpublished data suggest that other annealing helicases like Smarcal1 involved in resolving the chicken-foot structure [99,100] also interact with NEIL1 [101]. Repair synthesis and ligation to seal the nick is thus carried out by Polδ followed by Lig 1, likely via LP-BER rather than by the canonical BER enzymes Polβ and Lig 3α. Once the repair is completed, replication resumes after fork resolution. 

In oxidatively stressed cells, an alternative sliding clamp, Rad9–Rad1–Hus1 or 9-1-1, which is structurally similar to the replication clamp PCNA [102], has been suggested to play a role in BER. 9-1-1 is loaded onto DNA by an RF-C variant clamp loader containing Rad17. 9-1-1 stabilizes the replication fork through Chk1-mediated G2/M arrest [103] and recruitment of WRN helicase [104]. A Lu in collaboration with us showed that 9-1-1 stimulates NEIL1 in replication fork-mimicking substrates [105]. 9-1-1 also activates FEN-1 and Lig 1, suggesting its role in repair at stalled replication forks [106]. However, its precise role in RA-BER requires further investigation.

### 5.3. Backup Function of NEIL2 in Pre-Replicative BER 

As mentioned before, NEIL2, which also binds base lesions in ssDNA as NEIL1 but without S phase-specific activation, has been linked to repair during transcription [37]. Furthermore, NEIL2 deficiency alone does not inhibit DNA replication after oxidative stress, but enhances replication inhibition in NEIL1-deficient cells. This suggests that in the absence of NEIL1, NEIL2 may participate as a ‘relief-pitcher’ for RA-BER. The broad and overlapping substrate specificities of NEIL2 and NEIL1 ensure the ability of NEIL2 to provide the backup function when needed [37]. Furthermore, the recently characterized NEIL3 was also shown to be activated during S phase [107]. However, the substrate preference of NEIL3 has not been fully characterized. It is possible that NEIL3 is also involved in pre-replicative repair for a different set of oxidized bases. Future studies should examine combined NEIL deficiency in mouse models. 

## 6. Concluding Remarks

Every time a human cell divides, the cell’s most essential component, the genome, is at higher risk of damage. Oxidized base damage in template DNA at the replication fork would invariably cause mutations if left unrepaired, leading to daughter cells riddled with genetic errors. However, mammalian cells have developed ways to repair such damage in ssDNA prior to replication, utilizing NEIL1 in coordination with the replication machinery. Both the RA-BER pathways, template strand-associated pre-replicative and progeny strand-specific post-replicative BER play a critical role in preventing and/or reversing mutations. As described in this commentary, efficient and timely recognition of these lesions in the template strand before replicative synthesis is a critical prerequisite for efficient pre-replicative repair. Whereas partial reconstitution of damage recognition, stalling of Polδ at primer-template substrates, and repair involving the NEIL1 complex and replication proteins provided key molecular insights into the mechanism of such pre-replicative BER, complete reconstitution of RA-BER is inconceivable, particularly at the chromatin level, and would involve a large network of proteins and tightly controlled signaling involving multiple helicases. Ongoing studies focus on sequential reconstitution of the RA-BER steps in vitro using NEIL1 (or other DGs) complexes immunoprecipitated from human cells specifically deficient in the key helicases and replication proteins. These studies should shed light on the role of these proteins in the phenomenon of BER.

## Figures and Tables

**Table 1:** Common and unique substrates of mammalian DNA glycosylases (DGs).

Mammalian DG	Preferred Substrates
NEIL1	FapyA, FapyG, Tg, 5-OHC, 5-OHU, DHU, 5-formyl U, DHU, DHT, single stranded 8-oxo-G (oxo-G opposite C), hydantoin lesions, Gh, Sp, 5OHMH
NEIL2	5-OHC, 5-OHU, DHT, DHU, Tg, 8-oxo-G, Gh, Sp
NEIL3	FapyA, FapyG, Tg, 5-OHC, 5-OHU, DHU, DHT, Gh, Sp, 5OHMH
OGG1	8-oxo-G, 8-Oxo-A, Fapy G; prefers lesion opposite C
NTHL1	5-OHU, 5-OHC, TG, DHU, and FapyG

8-oxo-G: 8-oxo-7,8-dihydroguanine; 8-oxo-A: 7,8-dihydro-8-oxoadenine; FapyG: 2,6-diamino-4-hydroxy-5-formamidopyrimidine; FapyA: 4,6-diamino-5-formamidopyrimidine; MeFapyG: 2,6-diamino-4-hydroxy-5N-methylformamidopyrimidine; Sp: Spiroiminodihydantoin; Gh: guanidinohydantoin; Tg: thymine glycol; 5-OHC: 5-hydroxycytosine; 5-OHU: 5-hydroxyruacil; 5OHMH: 5-hydroxy-5-methylhydantion; DHT: 5,6-dihydrothymine; DHU: 5,6-dihydrouracil; 5-formyl U: 5-formyluracil.

**Table 2:** Association of *NEIL1* and *NEIL2* with single nucleotide polymorphisms (SNPs) and reported disease-associated risks.

Gene	SNP Database Entry Number	Reported Risks	References
*NEIL1*	rs4462560	Radiation-induced esophageal toxicity, depression disorders.	[60,61]
	rs5745908	Familial colorectal cancer.	[56]
*NEIL2*	rs804269, rs804268, rs8191613, rs8191642, rs8191663, rs8191664, rs1534862, rs8191667	Familial colorectal cancer.	[56]
	rs146678	Associated with breast cancer risk in BRCA2 mutation carriers.	[70]
	rs804270	Increased risk for gastric cancer, increased risk of squamous cell carcinomas of the oral cavity and oropharynx.	[59,71]

**Table 3:** Functional association of DNA replication proteins with NEIL1.

DNA Replication Proteins	Functional Association with NEIL1	Reference
PCNA	PCNA stimulates NEIL1 activity in excising 5-OHU from single-stranded DNA sequences, including fork structures. PCNA enhances NEIL1 loading on the substrate.	[36,38,39,82]
FEN-1	NEIL1 participates in strand displacement repair synthesis (LP-BER) mediated by FEN-1 and stimulated by PCNA. FEN-1 cleaves the 5′-overhanging flap structure that is generated by displacement synthesis when DNA polymerase encounters the 5′ end of a downstream Okazaki fragment.	[82]
RPA	RPA coats the ssDNA template at the replication fork and inhibits NEIL1’s activity (to regulate excision of oxidative DNA base damage in primer-template structures) via direct interaction, as shown through in vivo and in vitro analysis.	[81]
RF-C	RF-C activates NEIL1-initiated LP-BER along with DNA replication proteins as shown through in vivo and in vitro analysis.	[3]
Polδ	NEIL1 physically interacts with Polδ as shown by in vivo and in vitro analysis.	[3]
Lig 1	NEIL1 physically interacts with Lig 1 as shown by in vivo and in vitro analysis.	[3]
WRN	WRN stimulates NEIL1 to excise oxidative lesions from bubble DNA substrates.	[83]

PCNA: proliferating cell nuclear antigen; FEN-1: flap endonuclease 1; RPA: replication protein A; RF-C: replication factor C; Polδ: polymerase delta; Lig 1: ligase 1; WRN: Werner helicase.

## References

[1] Roos W.P., Thomas A.D., Kaina B. (2016). DNA damage and the balance between survival and death in cancer biology. Nat. Rev. Cancer.

[2] Maynard S., Fang E.F., Scheibye-Knudsen M., Croteau D.L., Bohr V.A. (2015). DNA Damage, DNA Repair, Aging, and Neurodegeneration. Cold Spring Harb. Perspect. Med..

[3] Hegde P.M., Dutta A., Sengupta S., Mitra J., Adhikari S., Tomkinson A.E., Li G.M., Boldogh I., Hazra T.K., Mitra S. (2015). The C-terminal Domain (CTD) of Human DNA Glycosylase NEIL1 Is Required for Forming BERosome Repair Complex with DNA Replication Proteins at the Replicating Genome: DOMINANT NEGATIVE FUNCTION OF THE CTD. J. Biol. Chem..

[4] Bjelland S., Seeberg E. (2003). Mutagenicity, toxicity and repair of DNA base damage induced by oxidation. Mutat. Res..

[5] Kreutzer D.A., Essigmann J.M. (1998). Oxidized, deaminated cytosines are a source of C --> T transitions in vivo. Proc. Natl. Acad. Sci. USA.

[6] Watson J. (2013). Oxidants, antioxidants and the current incurability of metastatic cancers. Open Biol..

[7] Scott T.L., Rangaswamy S., Wicker C.A., Izumi T. (2014). Repair of oxidative DNA damage and cancer: Recent progress in DNA base excision repair. Antioxid. Redox Signal..

[8] Friedberg E.C., Aguilera A., Gellert M., Hanawalt P.C., Hays J.B., Lehmann A.R., Lindahl T., Lowndes N., Sarasin A., Wood R.D. (2006). DNA repair: From molecular mechanism to human disease. DNA Repair.

[9] Banerjee A., Yang W., Karplus M., Verdine G.L. (2005). Structure of a repair enzyme interrogating undamaged DNA elucidates recognition of damaged DNA. Nature.

[10] Collins A.R., Cadet J., Moller L., Poulsen H.E., Vina J. (2004). Are we sure we know how to measure 8-oxo-7,8-dihydroguanine in DNA from human cells?. Arch. Biochem. Biophys..

[11] Shibutani S., Takeshita M., Grollman A.P. (1991). Insertion of specific bases during DNA synthesis past the oxidation-damaged base 8-oxodG. Nature.

[12] Shigenaga M.K., Aboujaoude E.N., Chen Q., Ames B.N. (1994). Assays of oxidative DNA damage biomarkers 8-oxo-2′-deoxyguanosine and 8-oxoguanine in nuclear DNA and biological fluids by high-performance liquid chromatography with electrochemical detection. Methods Enzymol..

[13] Lindahl T. (1993). Instability and decay of the primary structure of DNA. Nature.

[14] Hegde M.L., Banerjee S., Hegde P.M., Bellot L.J., Hazra T.K., Boldogh I., Mitra S. (2012). Enhancement of NEIL1 protein-initiated oxidized DNA base excision repair by heterogeneous nuclear ribonucleoprotein U (hnRNP-U) via direct interaction. J. Biol. Chem..

[15] Hegde M.L., Hazra T.K., Mitra S. (2010). Functions of disordered regions in mammalian early base excision repair proteins. Cell. Mol. Life Sci..

[16] Zhang L., Lu X., Lu J., Liang H., Dai Q., Xu G.L., Luo C., Jiang H., He C. (2012). Thymine DNA glycosylase specifically recognizes 5-carboxylcytosine-modified DNA. Nat. Chem. Biol..

[17] Krokan H.E., Drablos F., Slupphaug G. (2002). Uracil in DNA--occurrence, consequences and repair. Oncogene.

[18] Lu A.L., Li X., Gu Y., Wright P.M., Chang D.Y. (2001). Repair of oxidative DNA damage: Mechanisms and functions. Cell Biochem. Biophys..

[19] Hazra T.K., Izumi T., Boldogh I., Imhoff B., Kow Y.W., Jaruga P., Dizdaroglu M., Mitra S. (2002). Identification and characterization of a human DNA glycosylase for repair of modified bases in oxidatively damaged DNA. Proc. Natl. Acad. Sci. USA.

[20] Bandaru V., Sunkara S., Wallace S.S., Bond J.P. (2002). A novel human DNA glycosylase that removes oxidative DNA damage and is homologous to Escherichia coli endonuclease VIII. DNA Repair.

[21] Burrows C.J., Muller J.G., Kornyushyna O., Luo W., Duarte V., Leipold M.D., David S.S. (2002). Structure and potential mutagenicity of new hydantoin products from guanosine and 8-oxo-7,8-dihydroguanine oxidation by transition metals. Environ. Health Perspect..

[22] Bailly V., Verly W.G. (1987). Escherichia coli endonuclease III is not an endonuclease but a β-elimination catalyst. Biochem. J..

[23] Zharkov D.O., Golan G., Gilboa R., Fernandes A.S., Gerchman S.E., Kycia J.H., Rieger R.A., Grollman A.P., Shoham G. (2002). Structural analysis of an Escherichia coli endonuclease VIII covalent reaction intermediate. EMBO J..

[24] Wiederhold L., Leppard J.B., Kedar P., Karimi-Busheri F., Rasouli-Nia A., Weinfeld M., Tomkinson A.E., Izumi T., Prasad R., Wilson S.H. (2004). AP endonuclease-independent DNA base excision repair in human cells. Mol. Cell.

[25] Frosina G., Fortini P., Rossi O., Carrozzino F., Raspaglio G., Cox L.S., Lane D.P., Abbondandolo A., Dogliotti E. (1996). Two pathways for base excision repair in mammalian cells. J. Biol. Chem..

[26] Podlutsky A.J., Dianova I.I., Podust V.N., Bohr V.A., Dianov G.L. (2001). Human DNA polymerase β initiates DNA synthesis during long-patch repair of reduced AP sites in DNA. EMBO J..

[27] Busso C.S., Lake M.W., Izumi T. (2010). Posttranslational modification of mammalian AP endonuclease (APE1). Cell. Mol. Life Sci..

[28] Seet B.T., Dikic I., Zhou M.M., Pawson T. (2006). Reading protein modifications with interaction domains. Nat. Rev. Mol. Cell. Biol..

[29] Sengupta S.Y.C., Hegde M.L., Hegde P.M., Mitra J., Pandey A., Dutta A., Datarwala A.D., Bhakat K.K., Mitra S. (2017). Acetylation of NEIL1 is required for its repair complex formation in chromatin: Associated with resistance to oxidative stress. J. Biol. Chem..

[30] Bhakat K.K., Hazra T.K., Mitra S. (2004). Acetylation of the human DNA glycosylase NEIL2 and inhibition of its activity. Nucleic Acids Res..

[31] Prakash A., Cao V.B., Doublie S. (2016). Phosphorylation Sites Identified in the NEIL1 DNA Glycosylase Are Potential Targets for the JNK1 Kinase. PLoS ONE.

[32] Liu X., Roy R. (2002). Truncation of amino-terminal tail stimulates activity of human endonuclease III (hNTH1). J. Mol. Biol..

[33] Nilsen H., Otterlei M., Haug T., Solum K., Nagelhus T.A., Skorpen F., Krokan H.E. (1997). Nuclear and mitochondrial uracil-DNA glycosylases are generated by alternative splicing and transcription from different positions in the UNG gene. Nucleic Acids Res..

[34] Bhakat K.K., Izumi T., Yang S.H., Hazra T.K., Mitra S. (2003). Role of acetylated human AP-endonuclease (APE1/Ref-1) in regulation of the parathyroid hormone gene. EMBO J..

[35] Hegde M.L., Hegde P.M., Arijit D., Boldogh I., Mitra S. (2012). Human DNA Glycosylase NEIL1’s Interactions with Downstream Repair Proteins Is Critical for Efficient Repair of Oxidized DNA Base Damage and Enhanced Cell Survival. Biomolecules.

[36] Dou H., Theriot C.A., Das A., Hegde M.L., Matsumoto Y., Boldogh I., Hazra T.K., Bhakat K.K., Mitra S. (2008). Interaction of the human DNA glycosylase NEIL1 with proliferating cell nuclear antigen. The potential for replication-associated repair of oxidized bases in mammalian genomes. J. Biol. Chem..

[37] Hegde M.L., Hazra T.K., Mitra S. (2008). Early steps in the DNA base excision/single-strand interruption repair pathway in mammalian cells. Cell Res..

[38] Hegde M.L., Hegde P.M., Bellot L.J., Mandal S.M., Hazra T.K., Li G.M., Boldogh I., Tomkinson A.E., Mitra S. (2013). Prereplicative repair of oxidized bases in the human genome is mediated by NEIL1 DNA glycosylase together with replication proteins. Proc. Natl. Acad. Sci. USA.

[39] Prakash A., Moharana K., Wallace S.S., Doublie S. (2017). Destabilization of the PCNA trimer mediated by its interaction with the NEIL1 DNA glycosylase. Nucleic Acids Res..

[40] Prasad R., Dyrkheeva N., Williams J., Wilson S.H. (2015). Mammalian Base Excision Repair: Functional Partnership between PARP-1 and APE1 in AP-Site Repair. PLoS ONE.

[41] Hegde M.L., Tsutakawa S.E., Hegde P.M., Holthauzen L.M., Li J., Oezguen N., Hilser V.J., Tainer J.A., Mitra S. (2013). The disordered C-terminal domain of human DNA glycosylase NEIL1 contributes to its stability via intramolecular interactions. J. Mol. Biol..

[42] Szczesny B., Tann A.W., Longley M.J., Copeland W.C., Mitra S. (2008). Long patch base excision repair in mammalian mitochondrial genomes. J. Biol. Chem..

[43] Wilson S.H., Kunkel T.A. (2000). Passing the baton in base excision repair. Nat. Struct. Biol..

[44] Yang C., Sengupta S., Hegde P.M., Mitra J., Jiang S., Holey B., Sarker A.H., Tsai M.S., Hegde M.L., Mitra S. (2017). Regulation of oxidized base damage repair by chromatin assembly factor 1 subunit A. Nucleic Acids Res..

[45] Bohr V.A., Smith C.A., Okumoto D.S., Hanawalt P.C. (1985). DNA repair in an active gene: Removal of pyrimidine dimers from the DHFR gene of CHO cells is much more efficient than in the genome overall. Cell.

[46] Dutta A., Yang C., Sengupta S., Mitra S., Hegde M.L. (2015). New paradigms in the repair of oxidative damage in human genome: Mechanisms ensuring repair of mutagenic base lesions during replication and involvement of accessory proteins. Cell. Mol. Life Sci..

[47] Kadyrov F.A., Holmes S.F., Arana M.E., Lukianova O.A., O’Donnell M., Kunkel T.A., Modrich P. (2007). Saccharomyces cerevisiae MutLalpha is a mismatch repair endonuclease. J. Biol. Chem..

[48] Chakraborty A., Wakamiya M., Venkova-Canova T., Pandita R.K., Aguilera-Aguirre L., Sarker A.H., Singh D.K., Hosoki K., Wood T.G., Sharma G. (2015). Neil2-null Mice Accumulate Oxidized DNA Bases in the Transcriptionally Active Sequences of the Genome and Are Susceptible to Innate Inflammation. J. Biol. Chem..

[49] Klungland A., Rosewell I., Hollenbach S., Larsen E., Daly G., Epe B., Seeberg E., Lindahl T., Barnes D.E. (1999). Accumulation of premutagenic DNA lesions in mice defective in removal of oxidative base damage. Proc. Natl. Acad. Sci. USA.

[50] Minowa O., Arai T., Hirano M., Monden Y., Nakai S., Fukuda M., Itoh M., Takano H., Hippou Y., Aburatani H. (2000). Mmh/Ogg1 gene inactivation results in accumulation of 8-hydroxyguanine in mice. Proc. Natl. Acad. Sci. USA.

[51] Osterod M., Hollenbach S., Hengstler J.G., Barnes D.E., Lindahl T., Epe B. (2001). Age-related and tissue-specific accumulation of oxidative DNA base damage in 7,8-dihydro-8-oxoguanine-DNA glycosylase (Ogg1) deficient mice. Carcinogenesis.

[52] Gu H., Marth J.D., Orban P.C., Mossmann H., Rajewsky K. (1994). Deletion of a DNA polymerase β gene segment in T cells using cell type-specific gene targeting. Science.

[53] Xanthoudakis S., Smeyne R.J., Wallace J.D., Curran T. (1996). The redox/DNA repair protein, Ref-1, is essential for early embryonic development in mice. Proc. Natl. Acad. Sci. USA.

[54] Prakash A., Carroll B.L., Sweasy J.B., Wallace S.S., Doublie S. (2014). Genome and cancer single nucleotide polymorphisms of the human NEIL1 DNA glycosylase: Activity, structure, and the effect of editing. DNA Repair.

[55] Shinmura K., Tao H., Goto M., Igarashi H., Taniguchi T., Maekawa M., Takezaki T., Sugimura H. (2004). Inactivating mutations of the human base excision repair gene NEIL1 in gastric cancer. Carcinogenesis.

[56] Broderick P., Bagratuni T., Vijayakrishnan J., Lubbe S., Chandler I., Houlston R.S. (2006). Evaluation of NTHL1, NEIL1, NEIL2, MPG, TDG, UNG and SMUG1 genes in familial colorectal cancer predisposition. BMC Cancer.

[57] Goto M., Shinmura K., Tao H., Tsugane S., Sugimura H. (2010). Three novel NEIL1 promoter polymorphisms in gastric cancer patients. World J. Gastrointest. Oncol..

[58] Shinmura K., Kato H., Kawanishi Y., Igarashi H., Goto M., Tao H., Inoue Y., Nakamura S., Misawa K., Mineta H. (2016). Abnormal Expressions of DNA Glycosylase Genes NEIL1, NEIL2, and NEIL3 Are Associated with Somatic Mutation Loads in Human Cancer. Oxid. Med. Cell. Longev..

[59] Zhai X., Zhao H., Liu Z., Wang L.E., El-Naggar A.K., Sturgis E.M., Wei Q. (2008). Functional variants of the NEIL1 and NEIL2 genes and risk and progression of squamous cell carcinoma of the oral cavity and oropharynx. Clin. Cancer Res..

[60] Czarny P., Kwiatkowski D., Galecki P., Talarowska M., Orzechowska A., Bobinska K., Bielecka-Kowalska A., Szemraj J., Maes M., Su K.P. (2015). Association between single nucleotide polymorphisms of MUTYH, hOGG1 and NEIL1 genes, and depression. J. Affect. Disord..

[61] Chen Y., Zhu M., Zhang Z., Jiang G., Fu X., Fan M., Sun M., Wei Q., Zhao K. (2013). A NEIL1 single nucleotide polymorphism (rs4462560) predicts the risk of radiation-induced toxicities in esophageal cancer patients treated with definitive radiotherapy. Cancer.

[62] Modrich P., Lahue R. (1996). Mismatch repair in replication fidelity, genetic recombination, and cancer biology. Annu. Rev. Biochem..

[63] Lindahl T. (1974). An N-glycosidase from Escherichia coli that releases free uracil from DNA containing deaminated cytosine residues. Proc. Natl. Acad. Sci. USA.

[64] Arai T., Kelly V.P., Minowa O., Noda T., Nishimura S. (2006). The study using wild-type and Ogg1 knockout mice exposed to potassium bromate shows no tumor induction despite an extensive accumulation of 8-hydroxyguanine in kidney DNA. Toxicology.

[65] Chan M.K., Ocampo-Hafalla M.T., Vartanian V., Jaruga P., Kirkali G., Koenig K.L., Brown S., Lloyd R.S., Dizdaroglu M., Teebor G.W. (2009). Targeted deletion of the genes encoding NTH1 and NEIL1 DNA N-glycosylases reveals the existence of novel carcinogenic oxidative damage to DNA. DNA Repair.

[66] Krishnamurthy N., Zhao X., Burrows C.J., David S.S. (2008). Superior removal of hydantoin lesions relative to other oxidized bases by the human DNA glycosylase hNEIL1. Biochemistry.

[67] Parker J.B., Bianchet M.A., Krosky D.J., Friedman J.I., Amzel L.M., Stivers J.T. (2007). Enzymatic capture of an extrahelical thymine in the search for uracil in DNA. Nature.

[68] Slupphaug G., Mol C.D., Kavli B., Arvai A.S., Krokan H.E., Tainer J.A. (1996). A nucleotide-flipping mechanism from the structure of human uracil-DNA glycosylase bound to DNA. Nature.

[69] Mitra S., Izumi T., Boldogh I., Bhakat K.K., Hill J.W., Hazra T.K. (2002). Choreography of oxidative damage repair in mammalian genomes. Free Radic. Biol. Med..

[70] Osorio A., Milne R.L., Kuchenbaecker K., Vaclova T., Pita G., Alonso R., Peterlongo P., Blanco I., de la Hoya M., Duran M. (2014). DNA glycosylases involved in base excision repair may be associated with cancer risk in BRCA1 and BRCA2 mutation carriers. PLoS Genet..

[71] Elingarami S., Liu H., Kalinjuma A.V., Hu W., Li S., He N. (2015). Polymorphisms in NEIL-2, APE-1, CYP2E1 and MDM2 Genes are Independent Predictors of Gastric Cancer Risk in a Northern Jiangsu Population (China). J. Nanosci. Nanotechnol..

[72] Cowell I.G., Sunter N.J., Singh P.B., Austin C.A., Durkacz B.W., Tilby M.J. (2007). gammaH2AX foci form preferentially in euchromatin after ionising-radiation. PLoS ONE.

[73] Takata H., Hanafusa T., Mori T., Shimura M., Iida Y., Ishikawa K., Yoshikawa K., Yoshikawa Y., Maeshima K. (2013). Chromatin compaction protects genomic DNA from radiation damage. PLoS ONE.

[74] Berquist B.R., Wilson D.M. (2012). Pathways for repairing and tolerating the spectrum of oxidative DNA lesions. Cancer Lett..

[75] Odell I.D., Wallace S.S., Pederson D.S. (2013). Rules of engagement for base excision repair in chromatin. J. Cell. Physiol..

[76] Banerjee D., Mandal S.M., Das A., Hegde M.L., Das S., Bhakat K.K., Boldogh I., Sarkar P.S., Mitra S., Hazra T.K. (2011). Preferential repair of oxidized base damage in the transcribed genes of mammalian cells. J. Biol. Chem..

[77] Parlanti E., Locatelli G., Maga G., Dogliotti E. (2007). Human base excision repair complex is physically associated to DNA replication and cell cycle regulatory proteins. Nucleic Acids Res..

[78] Dou H., Mitra S., Hazra T.K. (2003). Repair of oxidized bases in DNA bubble structures by human DNA glycosylases NEIL1 and NEIL2. J. Biol. Chem..

[79] Fleming A.M., Burrows C.J. (2017). Formation and processing of DNA damage substrates for the hNEIL enzymes. Free Radic. Biol. Med..

[80] Zhou J., Fleming A.M., Averill A.M., Burrows C.J., Wallace S.S. (2015). The NEIL glycosylases remove oxidized guanine lesions from telomeric and promoter quadruplex DNA structures. Nucleic Acids Res..

[81] Theriot C.A., Hegde M.L., Hazra T.K., Mitra S. (2010). RPA physically interacts with the human DNA glycosylase NEIL1 to regulate excision of oxidative DNA base damage in primer-template structures. DNA Repair.

[82] Hegde M.L., Theriot C.A., Das A., Hegde P.M., Guo Z., Gary R.K., Hazra T.K., Shen B., Mitra S. (2008). Physical and functional interaction between human oxidized base-specific DNA glycosylase NEIL1 and flap endonuclease 1. J. Biol. Chem..

[83] Das A., Boldogh I., Lee J.W., Harrigan J.A., Hegde M.L., Piotrowski J., de Souza Pinto N., Ramos W., Greenberg M.M., Hazra T.K. (2007). The human Werner syndrome protein stimulates repair of oxidative DNA base damage by the DNA glycosylase NEIL1. J. Biol. Chem..

[84] Aladjem M.I. (2007). Replication in context: Dynamic regulation of DNA replication patterns in metazoans. Nat. Rev. Genet..

[85] Rampakakis E., Arvanitis D.N., Di Paola D., Zannis-Hadjopoulos M. (2009). Metazoan origins of DNA replication: Regulation through dynamic chromatin structure. J. Cell. Biochem..

[86] Sclafani R.A., Holzen T.M. (2007). Cell cycle regulation of DNA replication. Annu. Rev. Genet..

[87] Bell S.P., Dutta A. (2002). DNA replication in eukaryotic cells. Annu. Rev. Biochem..

[88] Dutta A., Bell S.P. (1997). Initiation of DNA replication in eukaryotic cells. Annu. Rev. Cell Dev. Biol..

[89] Hook S.S., Lin J.J., Dutta A. (2007). Mechanisms to control rereplication and implications for cancer. Curr. Opin. Cell Biol..

[90] Krasinska L., Besnard E., Cot E., Dohet C., Mechali M., Lemaitre J.M., Fisher D. (2008). Cdk1 and Cdk2 activity levels determine the efficiency of replication origin firing in Xenopus. EMBO J..

[91] Iyama T., Wilson D.M. (2013). DNA repair mechanisms in dividing and non-dividing cells. DNA Repair.

[92] Akbari M., Solvang-Garten K., Hanssen-Bauer A., Lieske N.V., Pettersen H.S., Pettersen G.K., Wilson D.M., Krokan H.E., Otterlei M. (2010). Direct interaction between XRCC1 and UNG2 facilitates rapid repair of uracil in DNA by XRCC1 complexes. DNA Repair.

[93] Otterlei M., Warbrick E., Nagelhus T.A., Haug T., Slupphaug G., Akbari M., Aas P.A., Steinsbekk K., Bakke O., Krokan H.E. (1999). Post-replicative base excision repair in replication foci. EMBO J..

[94] Matsumoto Y. (2001). Molecular mechanism of PCNA-dependent base excision repair. Prog. Nucleic Acid. Res. Mol. Biol..

[95] Jiricny J. (2013). Postreplicative mismatch repair. Cold Spring Harb. Perspect. Biol..

[96] Bjoras K.O., Sousa M.M.L., Sharma A., Fonseca D.M., Sogaard C.K., Bjoras M., Otterlei M. (2017). Monitoring of the spatial and temporal dynamics of BER/SSBR pathway proteins, including MYH, UNG2, MPG, NTH1 and NEIL1-3, during DNA replication. Nucleic Acids Res..

[97] Hazra T.K., Izumi T., Maidt L., Floyd R.A., Mitra S. (1998). The presence of two distinct 8-oxoguanine repair enzymes in human cells: Their potential complementary roles in preventing mutation. Nucleic Acids Res..

[98] Popuri V., Croteau D.L., Bohr V.A. (2010). Substrate specific stimulation of NEIL1 by WRN but not the other human RecQ helicases. DNA Repair.

[99] Postow L., Woo E.M., Chait B.T., Funabiki H. (2009). Identification of SMARCAL1 as a component of the DNA damage response. J. Biol. Chem..

[100] Driscoll R., Cimprich K.A. (2009). HARPing on about the DNA damage response during replication. Genes Dev..

[101] Hegde M.L., Mitra S. (2017).

[102] Bloom L.B. (2009). Loading clamps for DNA replication and repair. DNA Repair.

[103] Parrilla-Castellar E.R., Arlander S.J., Karnitz L. (2004). Dial 9-1-1 for DNA damage: The Rad9-Hus1-Rad1 (9-1-1) clamp complex. DNA Repair.

[104] Hustedt N., Gasser S.M., Shimada K. (2013). Replication checkpoint: Tuning and coordination of replication forks in s phase. Genes.

[105] Bermudez V.P., Lindsey-Boltz L.A., Cesare A.J., Maniwa Y., Griffith J.D., Hurwitz J., Sancar A. (2003). Loading of the human 9-1-1 checkpoint complex onto DNA by the checkpoint clamp loader hRad17-replication factor C complex in vitro. Proc. Natl. Acad. Sci. USA.

[106] Balakrishnan L., Brandt P.D., Lindsey-Boltz L.A., Sancar A., Bambara R.A. (2009). Long patch base excision repair proceeds via coordinated stimulation of the multienzyme DNA repair complex. J. Biol. Chem..

[107] Neurauter C.G., Luna L., Bjoras M. (2012). Release from quiescence stimulates the expression of human NEIL3 under the control of the Ras dependent ERK-MAP kinase pathway. DNA Repair.

